# Evaluating the Improvement in Colonoscopy Quality Indicators Subsequent to Publication of Professional Society Guidelines

**DOI:** 10.7759/cureus.13040

**Published:** 2021-01-31

**Authors:** Anshu Wadehra, Hamid-Reza Moein, Diana Kakos, Eskara Pervez, Salina Faidhalla, Heba Habbal, Hajra Khan, Mahvish Khalid, Paul Naylor, Bashar Mohamad

**Affiliations:** 1 Internal Medicine, Wayne State University/Detroit Medical Center, Detroit, USA; 2 Internal Medicine, Sinai-Grace Hospital/Detroit Medical Center, Detroit, USA; 3 Internal Medicine, University of Michigan, Ann Arbor, USA; 4 Internal Medicine, Beaumont Hospital, Dearborn, USA; 5 Gastroenterology, Wayne State University, Detroit, USA

**Keywords:** colonoscopy quality indicators, colon cancer screening, adenoma detection rate

## Abstract

Introduction

Quality metrics of colonoscopy should be routinely monitored with a focus on optimizing the patient’s subsequent risk of colorectal cancer development. Documentation of bowel preparation, adenoma detection rate (ADR), and post-colonoscopy follow-up recommendations are three of the most important quality indicators for colonoscopy, but significant improvement has been challenging to achieve. The goal of this study is to determine whether the publication of colonoscopy quality indicator guidelines in 2015 resulted in an improvement in quality measures of physicians in our endoscopy suite as compared to before.

Methods

We reviewed the electronic medical records of patients who underwent a screening or surveillance colonoscopy in 2014 and 2017. Colonoscopies were performed in an open-access medical center endoscopy suite, staffed by three groups of physicians (academic gastroenterologists (AGs), non-academic gastroenterologists (non-AGs), and surgeons). We gathered demographic data, bowel preparation reports, follow-up recommendations, and notice to patient’s primary care physician, and calculated ADR for patients. Age- and gender-matched patients in both years were analyzed for ADR. These data were further subcategorized for each group of physicians.

Results

There were 553 patients in 2014 and 1,095 in 2017. Overall, male gender and African American race constituted the majority of patients in both years. Among age- and gender-matched patients in 2014 and 2017 (412 and 243 patients, respectively), ADR within each group of endoscopists was not significantly different between these two years (AGs 44% vs. 50%; non-AGs 32% vs. 27%; surgeons 25% vs. 21%; p>0.05 for all). However, in 2014 and 2017, ADR was significantly higher in the AG group as compared to the non-AG group and surgeons (p<0.006 and p<0.0004, respectively). Reporting of bowel preparation quality (82% vs. 87%) and documenting the recommended period for follow-up surveillance colonoscopy in the report (68% vs. 78%) improved between 2014 and 2017 (p=0.002 and p=0.0001, respectively). Correct recommendations for follow-up surveillance colonoscopy only improved significantly in the AG group (74% in 2014 as compared with 82% in 2017, p=0.003).

Conclusion

Based on the current guidelines, AG physicians are far exceeding the target ADR goals, and are superior compared to other groups of endoscopists. Although improvements were noted after guideline publications, areas of needed improvement with respect to meeting gastroenterology society guidelines for quality remained. The fact that individual physicians are performing and billing in an endoscopy suite staffed and equipped by a medical center creates an environment where responsibility for improvement in quality cannot be readily assigned.

## Introduction

Colonoscopy is the gold standard to detect colorectal cancer (CRC) and to prevent or reduce the risk of CRC by removing adenomatous polyps [1]. Quality metrics of colonoscopy for screening and surveillance of CRC should be routinely monitored since failure to meet quality standards could impact on subsequent CRC development. Unfortunately, assessing, improving, and maintaining quality in colonoscopies performed in a medical center endoscopy suite with multiple independent users is complicated by the issue of responsibility for the performance of the colonoscopies.

Adenoma detection rate (ADR), bowel preparation, and recommendations for future colonoscopy screening are three critical quality measures in colonoscopy [2-4]. ADR is defined as the fraction of patients undergoing colonoscopy with at least one adenoma detected and by strict definition should be in patients undergoing screening as opposed to surveillance colonoscopies [5,6]. Since ADR is inversely correlated with CRC incidence and death, it is a critical quality assessment criterion [5,6]. The American Society for Gastrointestinal Endoscopy/American College of Gastroenterology Task Force recommended in 2015 that a new target ADR for screening colonoscopies of ≥30% for males, ≥20% for females, and ≥25% for males/females combined would serve as a key quality assessment marker [4].

Quality of bowel preparation also plays a major role in therapeutic safety and diagnostic accuracy of colonoscopy [3,7-13]. Inadequate bowel preparation can lead to prolonged procedure time, increased costs, adverse events, and an estimated adenoma miss rate of 28-42% [3,7]. According to the latest Standards of Practice Committee of the American Society for Gastrointestinal Endoscopy, repeat colonoscopy in less than one year is recommended for inadequate bowel preparations [8].

Consistent with the need to provide patients with a scientifically sound recommendation for follow-up colonoscopy, guidelines regarding this are also published [4,14-16]. Thus, as a part of quality assessment, the documentation of informing the patient of a follow-up recommendation and the compliance of the recommendation with guidelines is the third key quality indicator to be assessed in this study.

With respect to our population, which is predominately African American (AA), information in the AA patient population regarding these quality assessments is lacking in the literature. Given that AAs have the highest incidence and mortality rate from CRC in the United States [14], characterizing the ADR, bowel preparation quality, and documentation of correct intervals for follow-up in this patient population is of high importance.

In this study, we aim to evaluate quality guideline compliance among varying endoscopists prior to and after the advent of the updated quality indicators in 2015. Specifically, we assessed whether guidelines were being met and if there was an increase in ADR as a result of the awareness of the importance of ADR.

## Materials and methods

Study population and measurements

This was a retrospective, cross-sectional, chart review study in an open-access endoscopy suite at Harper University Hospital (Detroit Medical Center) affiliated with Wayne State University School of Medicine. Electronic medical records of patients 62 years or older who underwent screening or surveillance colonoscopy in 2014 and all patients over the age of 40 in the last six months of 2017 were reviewed. Age, gender, race, bowel preparation quality, adenoma detection based on pathology results, number and size of polyps, endoscopist specialty (academic gastroenterology faculty (AG), non-academic gastroenterologists (non-AGs), and surgeons), surveillance vs. screening colonoscopy, and follow-up recommendations were extracted from electronic medical records. Those patients with missing histologic data, adenocarcinoma, or those who had aborted procedures were excluded from the ADR calculations. In addition, for ADR comparisons (since age and gender are strong determinants of ADR), a subgroup of age- and gender-matched patients in both 2014 and 2017 were used for comparison assessments. Institutional Review Board approval was obtained from Wayne State University.

Colonoscopy and follow-up recommendations

All colonoscopies were performed in an open-access medical center endoscopy suite by certified AGs, non-AGs, or surgeons with or without the assistance of fellowship trainees. The medical center was responsible for providing the facility, equipment, and support personnel. The individual physicians were responsible for the procedure. The follow-up interval for next colonoscopy was extracted based on the recommendations provided by the endoscopists in their procedure report and/or subsequent letter to the patient’s referring physician. The follow-up recommendations were compared to the guidelines by the United States Multi-Society Task Force on Colorectal Cancer, based on number of polyps or adenomas and quality of bowel preparation [4,15-17]. In brief, follow-up colonoscopy is recommended in less than a year for inadequate bowel preparation, in three to five years for adenomas based on their size and number, and in 10 years for hyperplastic or benign polyps.

Adenoma detection rate

Adenoma detection rate is defined as the proportion of screening colonoscopies with at least one adenoma detected. For comparison purposes, we also calculated ADR for surveillance colonoscopies, although this is not strictly adherent to the definition. Odds ratios (ORs) and 95% confidence intervals (CIs) were preferentially used for the comparisons of ADR.

Bowel preparation

Bowel preparation was similar among all patients with standard one dose (4 liters) of polyethylene glycol the day before colonoscopy. Bowel preparation was recorded based on the physician reporting in the procedure notes. Most endoscopists use Aronchick bowel preparation scale to describe the quality of bowel preparation (excellent, good, fair, and poor) [17].

Statistical analysis

Numeric data were presented as mean ± standard error of mean. ORs and CIs of 95% were used for presentation of the ADR. Pearson’s chi-square test and analysis of variance were used to compare the quality of bowel preparation with other variables. Linear regression and Spearman's test were used for correlations between bowel preparation and ADR with demographics. A p value of <0.05 is considered statistically significant. JMP 14.0 Software (SAS Institute Inc., Cary, NC, USA) was used for statistical analysis.

## Results

Demographics

We analyzed 553 patients in the 2014 group and 1,095 patients in the 2017 group. The entire group was used for assessment of colonoscopy quality measures with exception of the ADR, which utilized a subset of age- and gender-matched patients undergoing screening or surveillance colonoscopies between the age of 62 and 75 (Tables 1, 2). African Americans constituted 75% to 88%, in 2014 and 2017, respectively, of the studied patients, and the gender was similar between the groups. Most patients were seen for screening (75% in 2014 and 80% in 2017; p=0.01). The majority of colonoscopies were performed by gastroenterologists (1,060 patients by AGs (64%), 324 patients by non-AGs (20%), and 264 patients by surgeons (16%)).

**Table 1:** Demographic data of all patients AA, African American

Variables	2014 (n=553)	2017 (n=1095)	p-Value
Age (years)	66	59	0.0001
Gender (male)	46%	46%	0.97
Race (AA)	75%	88%	0.0001

**Table 2:** Demographic data of age- and gender-matched patients AA, African American

Variables	2014 (n=412)	2017 (n=243)	p-Value
Age (years)	66	66	0.76
Gender (male)	46%	44%	0.54
Race (AA)	75%	86%	0.0001

Adenoma detection rate among gastroenterologists and surgeons

In both 2014 and 2017, ADR was significantly higher in the AG group as compared to non-AGs and surgeons when evaluated in age- and gender-matched screening colonoscopy patients (p=0.006 and p=0.0004, in 2014 and 2017, respectively). When evaluated for change between 2014 and 2017, there was an increase in AG’s ADR and a decrease in non-AGs and surgeons (Table 3). Overall, however, there was no statistical difference for the three groups of endoscopists. To minimize the role of operator-dependent bias and due to the fact that some endoscopists may have joined or left our institution between 2014 and 2017, ADR was evaluated for physicians who performed at least five colonoscopies in both 2014 and 2017. The ADR trended similarly for all three groups, suggesting that a change in personnel did not account for ADR differences among different groups of endoscopists (data not shown).

**Table 3:** ADR for screening colonoscopies by physician speciality Academic GI, academic gastroenterologist; Non-academic GI, non-academic gastroenterologist; ADR, adenoma detection rate

Variables	ADR in 2014 (n=412) (%)	ADR in 2017 (n=243) (%)	Odds ratio	p-Value
Academic GI	44	50	0.79	0.267
Non-academic GI	32	27	1.27	0.5246
Surgeons	25	21	1.29	0.6055

Colonoscopy quality indicators

Colonoscopy quality measures are reported in the entire study population as well as a subset of age- and gender-matched patients undergoing screening colonoscopy (Tables 4, 5). The bowel preparation was reported in 82% of the patients in 2014 and improved to 87% in 2017 (p=0.002). A recommended interval for return colonoscopy was in the report for 68% in 2014 and it improved to 78% in 2017 (p=0.0001). The overall screening adenoma rate for all colonoscopies performed in the suite did not change significantly between 2014 and 2017 (38% vs. 35%, p=0.29). The same was true for the surveillance ADR (54% vs. 45%, p=0.097). When the evaluation was performed using the age- and gender-matched patients, a similar result was found in regard to the reporting of bowel preparation and screening ADR. However, reporting of the follow-up colonoscopy interval showed no significant change.

**Table 4:** Change in guideline-relevant quality parameters between 2014 and 2017 among all patients

Variables	2014 (n=553) (%)	2017 (n=1,095) (%)	Odds ratio	p-Value	Guidelines change
Bowel preparation quality reported	82	87	1.53	0.0026	Improved
Interval recommended in report	68	78	1.65	0.0001	Improved
Screening adenoma detection rate	38	35	1.14	0.297	No change
Surveillance adenoma detection rate	54	45	1.43	0.0965	No change

**Table 5:** Change in guideline-relevant quality parameters between 2014 and 2017 among age- and gender-matched patients

Variables	2014 (n=412) (%)	2017 (n=243) (%)	Odds ratio	p-Value	Guidelines change
Bowel preparation quality reported	82	88	1.57	0.0161	Improved
Interval recommended in report	68	72	1.21	0.2022	No change
Screening adenoma detection rate	38	40	0.93	0.532	No change

Compliance of follow-up recommendations with standard guidelines

Tables 6, 7 present the information with respect to guideline recommendations by the three groups of physicians performing colonoscopies. The colonoscopy report was sent to a primary physician more frequently in both years (2014 and 2017) in the AG group (93% and 88%, respectively) as compared to non-AGs (45% and 54%, respectively) or surgeons (41% and 4%, respectively). With respect to report of bowel preparation quality, AGs were again significantly better than the other two groups, although there was improvement noted for both non-AGs and surgeons. Academic gastroenterologists had the highest reporting of follow-up periods in their reports as compared to other two groups. Improvement in reporting the follow-up periods was observed for both non-AGs (38% to 59%; p=0.0002) and surgeons (14% to 39%; p=0.0001).

With respect to whether the reported recommendation was consistent with the guidelines, AGs were more likely than non-AGs and surgeons to be consistent with the guidelines. Academic gastroenterologists were the only group to have a statistically significant increase in the correct follow-up recommendation reported. When evaluated for whether the incorrect follow-up period was more likely to be longer or shorter, compared to the guideline recommendations, both the non-AGs and surgeons were more likely to error in recommending a shorter time to follow-up colonoscopy as compared to the AGs.

**Table 6:** Report of change between 2014 and 2017 in various quality measures PCP, primary care physician; Academic GI, academic gastroenterologist; Non-academic GI, non-academic gastroenterologist

Variables	Academic GI (%)		p-Value	Non-academic GI (%)		p-Value	Surgeons (%)		p-Value
	2014	2017		2014	2017		2014	2017	
PCP notified	93	88	0.042	45	54	0.088	41	4	0.0001
Bowel preparation quality reported	98	96	0.8852	59	75	0.0198	50	60	0.1266
Follow-up interval recommended in report	95	93	0.0418	38	59	0.0002	14	39	0.0001

**Table 7:** Report of change between 2014 and 2017 in follow-up interval recommendation compared to published guidelines Academic GI, academic gastroenterologist; Non-academic GI, non-academic gastroenterologist

Variables	Academic GI (%)		p-Value	Non-academic GI (%)		p-Value	Surgeons (%)		p-Value	Overall p-value for 2014 between all specialties	Overall p-value for 2017 between all specialties
	2014	2017		2014	2017		2014	2017			
Follow-up interval recommendation correctly follows guidelines	74	82	0.003	69	59	0.198	42	25	0.24	0.04	0.0001
Follow-up recommendation longer than guidelines	14	3		17	3		0	7			
Follow-up recommendation shorter than guidelines	12	15		13	39		58	68			

## Discussion

This study tested the hypothesis that colonoscopy quality measures would improve in the same endoscopy suite following the publication of clear guidelines. Our results were mixed, but overall there was minimal improvement in areas where improvement opportunities were present.

Based on the guidelines, academic gastroenterology physicians in the Detroit Medical Center Harper University Hospital endoscopy suite are far exceeding the target ADR goal of ≥25% for males/females combined and are superior, compared to other endoscopist groups, in the detection of adenomas. While non-AGs and surgeons had lower ADR, they were still close to the target rate as per the guidelines. While the high ADR for AGs might approach the maximum achievable for the population, improvement in non-AGs and surgeons after publication of the guidelines in which the importance of ADR and specific targets for ADR were emphasized was anticipated, but did not occur. The reason for this failure to improve was not readily assessable.

The issue of following colon polyp surveillance guidelines has been addressed previously by a number of authors [18,19]. One of the most informative studies was performed using a national survey questionnaire of gastroenterologists preparing for 2004 recertification [18]. The focus of the study was to determine whether there was a lack of knowledge or simply a personal disagreement with the guidelines. In that survey, only 57% reported that the guidelines were “very influential” in their practice. More significantly, up to 76% disagreed with the recommendation and chose to perform surveillance sooner than recommended. With respect to actual performance, a meta-analysis was recently published, which evaluated 16 studies [19]. The mean colonoscopy surveillance interval adherence rate was in the 50% range with respect to North American or European guidelines. The results of the gastroenterologists in this study are considerably better than 50% while that of the surgeons are lower. Among the reasons cited in the manuscript for non-adherence to guidelines included disagreement with the guidelines, concerns about missed polyps, poor bowel preparation, malpractice concerns, and reimbursement or monetary reasons. The overall outcomes due to this non-awareness of current guidelines and disagreement with guidelines were difficult to determine from the manuscripts reviewed.

A significant problem with respect to quality improvement assessments such as ours is that many endoscopy suites are provided as a service by medical centers, but there is no quality control responsibility by the center. Thus, individual physicians are responsible for their own quality assessments and improvements. Therefore, it is conceivable that the two groups with the lower ADR, which at least approaches the guidelines, are not aware that it is possible for them to achieve a higher ADR, as demonstrated by the AGs. Unless the medical center takes responsibility for posting quality guidelines and measuring the rates of achievement, it is unlikely that improvement will occur.

Our study had several possible deficiencies. With respect to sending a report to the primary care physician, the electronic medical record often did not have a primary care physician listed as the referring physician, so it was not possible to send the records to the appropriate personnel. Also, especially in the case of private practice gastroenterologists and surgeons, many patients may have a colonoscopy by the same physician who recommended the procedure, since they may see patients in their office before scheduling the procedure. With respect to bowel preparation, the fact that many physicians did not report the quality of bowel preparation in their report means that to some degree the role of bowel preparation and recommended follow-up may be inaccurate.

A strength of this study is that patient populations and bowel preparations are similar for all colonoscopies in the suite for the two time periods (2014 and 2017), so direct comparisons between groups can be made and improvements, as a result of guideline publication in 2015, could be accurately evaluated. This comparative study is unique since it uses a benchmark to assess compliance by comparing before and after the issuance of consensus guidelines on the quality of colonoscopy. The study is also more contemporary than the meta-analysis, which reported on manuscripts reviewing colonoscopies relying on guidelines issued between 2000 and 2012.

## Conclusions

Based on our observations, physicians’ recommendations for follow-up need to be stressed, especially with respect to recommending shorter interval for patients with minimal adenomas, which results in more frequent procedures than necessary. Also disturbing are the instances of failure to recommend a repeat procedure within a year for inadequate bowel preparations to minimize the risk of missing CRCs.

Finally, given the relatively low ADR for non-AGs and surgeons, as compared to AGs, it would seem important for the quality control managers of endoscopy suites to initiate procedures to help increase the ADR of endoscopists. Unfortunately, while areas of needed improvement with respect to meeting gastroenterology society guidelines for quality have been identified and were not significantly improved after publication of guidelines, the fact that individual physicians are performing and billing in an endoscopy suite staffed and equipped by a medical center creates an environment where responsibility for improvement in quality cannot be readily assigned.

## References

[1] Winawer SJ, Zauber AG, Ho MN (1993). Prevention of colorectal cancer by colonoscopic polypectomy. The National Polyp Study Workgroup. N Engl J Med.

[2] Gurudu SR, Ramirez FC (2013). Quality metrics in endoscopy. Gastroenterol Hepatol (N Y).

[3] Rex DK, Petrini JL, Baron TH (2006). Quality indicators for colonoscopy. Am J Gastroenterol.

[4] Rex DK, Schoenfeld PS, Cohen J (2015). Quality indicators for colonoscopy. Am J Gastroenterol.

[5] Corley DA, Jensen CD, Marks AR (2014). Adenoma detection rate and risk of colorectal cancer and death. N Engl J Med.

[6] Kaminski MF, Wieszczy P, Rupinski M (2017). Increased rate of adenoma detection associates with reduced risk of colorectal cancer and death. Gastroenterology.

[7] Saltzman JR, Cash BD, Pasha SF (2015). Bowel preparation before colonoscopy. Gastrointest Endosc.

[8] Levin B, Lieberman DA, McFarland B (2008). Screening and surveillance for the early detection of colorectal cancer and adenomatous polyps, 2008: a joint guideline from the American Cancer Society, the US Multi-Society Task Force on Colorectal Cancer, and the American College of Radiology. CA Cancer J Clin.

[9] Guo R, Wang YJ, Liu M (2019). The effect of quality of segmental bowel preparation on adenoma detection rate. BMC Gastroenterol.

[10] Clark BT, Rustagi T, Laine L (2014). What level of bowel prep quality requires early repeat colonoscopy: systematic review and meta-analysis of the impact of preparation quality on adenoma detection rate. Am J Gastroenterol.

[11] Calderwood AH, Thompson KD, Schroy 3rd PC, Lieberman DA, Jacobson BC (2015). Good is better than excellent: bowel preparation quality and adenoma detection rates. Gastrointest Endosc.

[12] Menees SB, Kim HM, Elliott EE, Mickevicius JL, Graustein BB, Schoenfeld PS (2013). The impact of fair colonoscopy preparation on colonoscopy use and adenoma miss rates in patients undergoing outpatient colonoscopy. Gastrointest Endosc.

[13] Larsen M, Hills N, Terdiman J (2011). The impact of the quality of colon preparation on follow-up colonoscopy recommendations. Am J Gastroenterol.

[14] Augustus GJ, Ellis NA (2018). Colorectal cancer disparity in African Americans: risk factors and carcinogenic mechanisms. Am J Pathol.

[15] Lieberman DA, Rex DK, Winawer SJ, Giardiello FM, Johnson DA, Levin TR (2012). Guidelines for colonoscopy surveillance after screening and polypectomy: a consensus update by the US Multi-Society Task Force on Colorectal Cancer. Gastroenterology.

[16] Aronchick CA, Lipshutz WH, Wright SH, Dufrayne F, Bergman G (2000). A novel tableted purgative for colonoscopic preparation: efficacy and safety comparisons with colyte and fleet phospho-soda. Gastrointest Endosc.

[17] Lai EJ, Calderwood AH, Doros G, Fix OK, Jacobson BC (2009). The Boston bowel preparation scale: a valid and reliable instrument for colonoscopy-oriented research. Gastrointest Endosc.

[18] Saini SD, Nayak RS, Kuhn L, Schoenfeld P (2009). Why don't gastroenterologists follow colon polyp surveillance guidelines?: results of a national survey. J Clin Gastroenterol.

[19] Djinbachian R, Dubé AJ, Durand M, Camara LR, Panzini B, Bouchard S, Von Renteln D (2019). Adherence to post-polypectomy surveillance guidelines: a systematic review and meta-analysis. Endoscopy.

